# Inter-city firm connections and the scaling of urban economic indicators

**DOI:** 10.1093/pnasnexus/pgae503

**Published:** 2024-11-09

**Authors:** Vicky Chuqiao Yang, Jacob J Jackson, Christopher P Kempes

**Affiliations:** MIT Sloan School of Management, Massachusetts Institute of Technology, Cambridge, MA 02142, USA; Institute for Data, Systems, and Society, Massachusetts Institute of Technology, Cambridge, MA 02142, USA; Santa Fe Institute, Santa Fe, NM 87501, USA; Department of Physics, Brown University, Providence, RI 02912, USA; Santa Fe Institute, Santa Fe, NM 87501, USA

## Abstract

Cities exhibit consistent returns to scale in economic outputs, and urban scaling analysis is widely adopted to uncover common mechanisms in cities’ socioeconomic productivity. Leading theories view cities as closed systems, with returns to scale arising from intra-city social interactions. Here, we argue that the interactions between cities, particularly via shared organizations such as firms, significantly influence a city’s economic output. By examining global data on city connectivity through multinational firms alongside urban scaling Gross Domestic Product (GDP) statistics from the United States, EU, and China, we establish that global connectivity notably enhances GDP, while controlling for population. After accounting for global connectivity, the effect of population on GDP is no longer distinguishable from linear. To differentiate between local and global mechanisms, we analyzed homicide case data, anticipating dominant local effects. As expected, inter-city connectivity showed no significant impact. Our research highlights that inter-city effects affect some urban outputs more than others. This empirical analysis lays the groundwork for incorporating inter-city organizational connections into urban scaling theories and could inform future model development.

**Significance Statement**

Leading urban scaling theories, which aim to explain the increased return to scale for socioeconomic outputs in cities, traditionally view cities as closed systems. By analyzing data across the United States, EU, and China, we demonstrate that inter-city connections, particularly through multinational firms, are associated with greater economic outputs, after controlling for population. While such connectivity boosts Gross Domestic Product (GDP), it does not similarly impact all urban phenomena, as illustrated by our analysis of homicide rates. Our findings underscore the importance of integrating both local and global interactions into urban scaling models, catalyzing a rethinking for the underlying mechanisms driving the superlinear scaling of urban economic outputs.

## Introduction

Throughout human history, the global urban population has grown continuously. More than half of the global population is currently urbanized, placing cities at the center of human development (1). Previous research has demonstrated power-law-like relationships between urban population (also referred to as size later in the text) and many urban outputs such as Gross Domestic Product (GDP), patents, violent crime, and contagious diseases that persist globally, including in the United States, EU, China, India, and Brazil (2–6). These relationships are described by


(1) $$Y(N)=Y_{0}N^{\beta },$$

where *Y* is an urban output, such as GDP or the number of crime instances, *N* is the population of the city, which spans several orders of magnitudes, $Y_{0}$ is a constant, and *β* is the scaling exponent. For many urban outputs, the scaling exponent *β* is greater than 1, suggesting greater rates of productivity in more populated cities. These observations, referred to as urban scaling, suggest the possibility of universal mechanisms influencing a wide variety of urban features across cities, despite differences in their individual characteristics such as geography and culture (2, 7–9). Understanding these mechanisms has important implications for developing more prosperous and safer cities.

The empirical observations have motivated the development of quantitative and mechanistic models for the origin of these scaling relationships. Most models, while varying in mathematical form, consider the superlinear scaling of socioeconomic outputs as a result of interactions among individuals within each city, typically attributed to densifying social connections in larger cities (7, 10, 11), or the resulting increased complementarity among diverse functions (12, 13) (see Ref. (14) for a review of urban scaling models).

However, cities do not exist in isolation, and a city’s economic output is necessarily affected by connections with other cities. In the words of Jacobs, “In modern and historical times… no city economy seems to have grown in isolation from other cities” (15). This idea is echoed in later studies, such as multinational corporations creating a “new international division of labor” (16). Connection with other cities is also observed to have complex implications on cities’ economic outcomes (17, 18). A minority of studies in the urban scaling framework suggest that *inter-city* mechanisms contribute to the superlinear scaling of urban outputs. For instance, Refs. (19, 20) demonstrate the link between population distribution across cities and GDP scaling exponents. Similarly, Ref. (21) hints at inter-city connections explaining scaling outliers like London, and Ref. (22) notes that incoming commuters are positively associated with wealth creation. However, inter-city effects have not been formally integrated into urban scaling theories. Only recently, Ref. (23) infers the degree of inter-city connections by assuming individual interactions across cities and fitting parameters from observed scaling relationships, and Ref. (24) studies how intercity communication and mobility networks are associated with patent activities.

In order to formulate a formal account of how inter-city connections affect urban scaling relationships, we need to first identify what kind of connections between cities are important for economic outputs. While most urban scaling models consider person-to-person interactions, it has long been known that economic performance is shaped by the kind and quantity of institutions—the constraints that shape what interactions are possible among individuals (25). Not all types of interactions lead to productive economic outcomes, which require the appropriate kind of organizations. This perspective is echoed in the context of cities. According to Sassen (26), cities are major nodes in the flow of information and wealth, which are key drivers of economic productivity, and these flows are intimately related to specialized business organizations, global service firms. Examples of these firms include financial institutions, consulting firms, accounting firms, law firms, and media organizations. Later studies built on this concept and quantified connections between cities using the co-presence of global service firms (27).

In this article, we provide a quantitative account of how metrics of inter-city connectivity through firms are associated with urban scaling relationships. Using a network of global cities based on the co-presence of multinational firms, GDP, and population data in the United States, EU, and China, we first show that cities more connected than expected of their size also over-perform the scaling relationship for GDP with city size. Second, we compare an urban scaling model which includes the global network connectivity, with the null model (equation (1)), and show that global network connectivity plays a significant role in predicting cities’ GDP production after accounting for city population. We also show that a model incorporating both variables better represents the data than the null model. We then discuss possible avenues for understanding the mechanisms for inter-city connections to affect a city’s economic outputs.

It is important to note that both inter- and intra-city connections are plausible mechanisms for superlinear scaling. One way to disambiguate these mechanisms is to investigate urban features that should and should not depend on inter-city firm connections. Given the importance of institutions discussed above, we expect GDP to depend on firm connections, but this should not be true for urban aspects such as homicide instances, which should be mostly subject to local effects. We repeat the analysis on homicide instances, which show no impact of global firm connectivity. Thus, economic superlinear scaling appears to depend on inter-city firm connectivity, while the superlinear scaling of homicide instances depends only on the increasing interactions of people within a city.

## Results

### Data

**Fig. 1.**
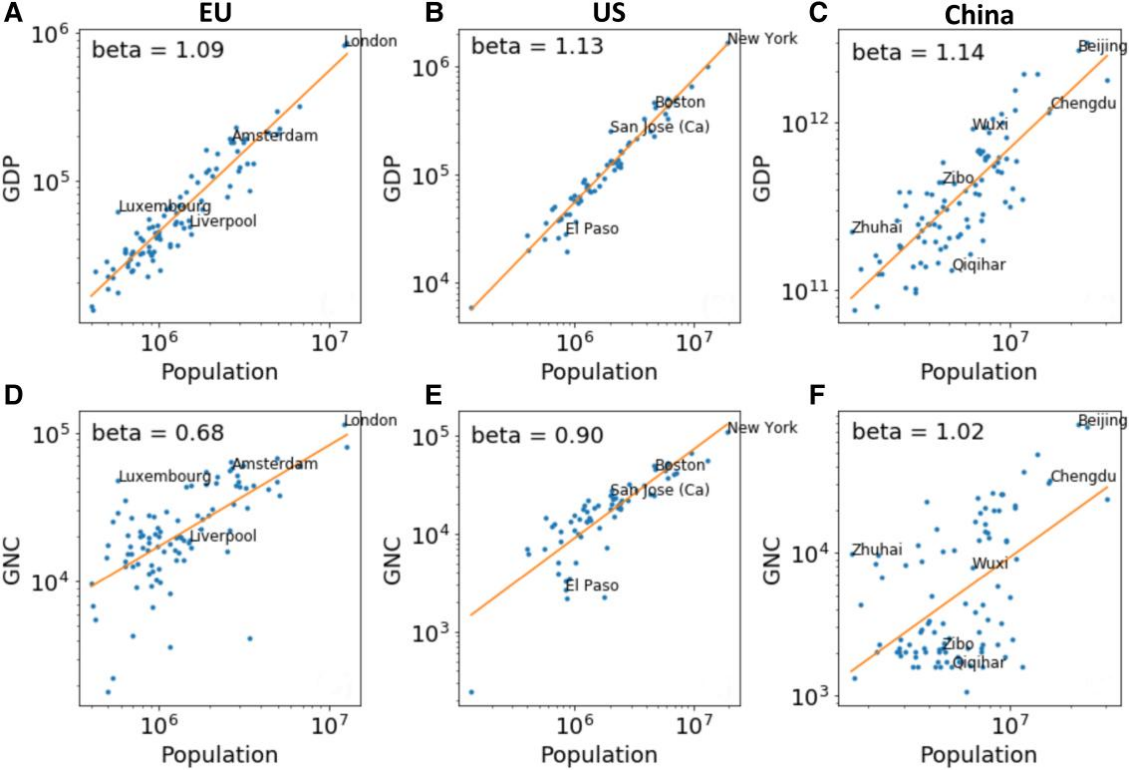
The scaling of GDP (top row) and GNC (bottom row) for the three regions of study in our project, EU (left column), United States (middle column), and China (right column). Selected cities are labeled for illustration.

Motivated by the important role of institutions in urban economic productivity, we measure connectivity between cities using the Global Network Connectivity (GNC) developed by Taylor and Derudder (27) (see Supplementary material for more detail). GNC uses the co-presence of 175 global firms to construct a network of 707 cities in the world. In this network, nodes are cities, and the edges between each pair of cities are weighted based on firm co-presence. When a firm has offices in both cities, it adds to the edge weight, with headquarters contributing more significantly than local offices. The GNC of a city is measured by its network degree, defined as the sum of all edge weights connected to that city. Besides the GNC data, we also use data on GDP and population for 94 cities in Europe, 66 cities in the United States, and 95 cities in China to perform scaling analysis. We chose these cities because they are the largest sample we have access to in these counties that overlap with cities available in the GNC dataset. The population data measures resident population in corresponding urban areas (see Supplementary material for more detail). The relationships between GDP and population in these three regions are shown in the top row of Fig. 1. We observe superlinear scaling in all three data sets, consistent with previous literature. The scaling exponents are 1.09 for the EU, 1.13 for the United States, and 1.14 for China. As shown in the bottom row of Fig. 1, GNC and population are positively, though not perfectly, correlated—that larger cities tend to have greater connectivity. The Pearson correlation between log population and log GNC is higher for the United States (0.82), while lower for the EU (0.65) and China (0.54).

Due to the scale effect of GNC, we will first perform a comparison of scaling residuals between GDP and GNC, which would eliminate any confounding effect of scale. That is, to show a city that over-performs in GNC as expected of its size also over-performs in GDP. We then perform a second regression analysis, where we compare a model that accounts for both GNC and population with one that considers population alone.

### Comparing scaling residuals of GDP and GNC

We compute and analyze the Scale-Adjusted Metropolitan Indicators (SAMIs) (28) for both GNC and GDP in each region of study, that is, to measure how much each city over and under-performs the scaling curve. The SAMI is meant for cross-comparison of indicators with a scale-adjusted metric, which accounts for differences in agglomeration effects between cities.

For city *i*, the SAMI for an urban indicator $Y_{i}$ (such as GNC or GDP) is, $\eta_{i}=\text{log}Y_{i}-\text{log}Y(N_{i})$, where $Y_{i}$ is the value in the data, $Y(N_{i})$ is the value expected of that city given its population $N_{i}$ from the scaling relation in equation (1).

**Fig. 2.**
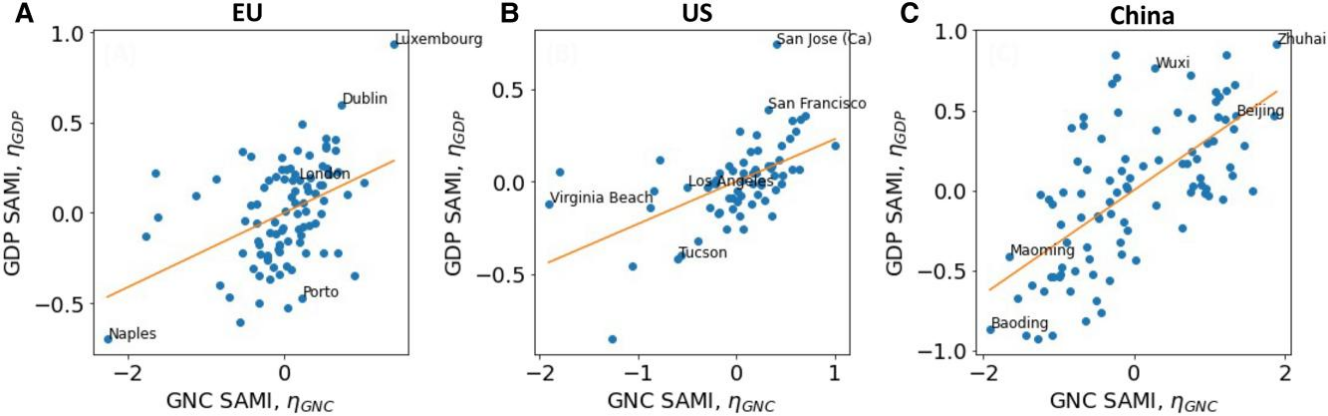
The relationship between SAMIs of GDP and that of GNC for (A) EU cities (Pearson correlation 0.42), (B) US cities (Pearson correlation 0.55), and (C) Chinese cities (Pearson correlation 0.66). Selected cities are labeled for illustration.

Figure 2 shows the relationship between the SAMIs of GNC and that of GDP for cities in the EU (A), United States (B), and China (C). In all three datasets, the two SAMIs are positively correlated. Pearson correlations are 0.42, 0.55, and 0.66 for the EU, United States, and China, respectively. All three correlations are statistically significant with p≤0.001. Table 1 summarizes the statistics of these correlations. Cities that over-perform in GNC than expected of their population also over-perform in GDP. These results suggest a city being more connected to other cities globally has a positive association with greater GDP production, after accounting for the scaling effects of cities.

**Table 1.** Statistics for the relationships between the SAMIs of GDP, $\eta_{\mathrm{GDP}}$, and those of GNC, $\eta_{\mathrm{GNC}}$ in three regions.

	No. of obs	Pearson corr	$R^{2}$	Slope
EU	94	0.42	0.18	0.21
United States	66	0.55	0.30	0.23
China	95	0.66	0.44	0.33

Slope ///denote the parameter in the linear fit $\eta_{\mathrm{GDP}}=\text{Slope}\;\eta_{\mathrm{GNC}}$. For all Pearson correlations reported, P≤0.001.

### Regression analysis

Besides relating the SAMIs of GDP and GNC, we estimate the parameters of logarithmic regression model that takes both population and GNC into account, and compare its performance with that of the null urban scaling model.

For data of each region (EU, United States, China), we perform the following regression


(2) logY=βlogN+αlogGNC+c.

We choose this model because we consider the effect of size and connectivity should interact in a multiplicative fashion—traditional urban scaling considers the economic output to be proportional to interactions in the form of $Y=c_{1}N^{\beta }$. Here, we consider what is produced through each interaction to be affected by GNC—those interactions related to global organizations have a greater economic impact than those do not, $Y=c_{2}N^{\beta }{\mathrm{GNC}}^{\alpha }$. Note that this form represents the Cobb–Douglas production function, typically used in Economics to represent how an output depends on multiple inputs. This model assumes each urban outputs to benefit from both intra- and inter-city city components, weighted by the exponents *β* and *α*, respectively. Taking log of both sides gives equation (2). We then estimate the parameters *β*, *α*, and constant *c*. We also compare this model with the null urban scaling model (from taking the logarithm of equation (1)),


(3) logY=βlogN+c,

by analyzing the coefficients and goodness of fit of the two models while accounting for the differences in model parameters.

The results for comparing the two models are summarized in Table 2, which contains key coefficients of both models and their 95% CI. In all three datasets, the coefficient for GNC, *α*, is significantly greater than zero, suggesting GNC plays an important role in predicting GDP even after taking population into account, echoing the finding in the SAMI analysis. Table 2 also shows model selection metrics, Adjusted R-square, Akaike Information Criterion (AIC), and Bayesian Information Criterion (BIC) for both models. These metrics evaluate the goodness of fit of the model to the data, while penalizing models with more parameters. A model fitting the data better would be reflected in a higher adjusted R-square, or lower AIC or BIC. We find that for all three datasets analyzed, the GNC model over-performs the null model on all three model selection metrics. This suggests the global connectivity of a city plays a significant role in the scaling relationship of the urban indicators.

**Table 2.** Estimated coefficients, their corresponding 95% CI, and model evaluation metrics for the GNC and null models.

	EU (94 observations)		United States (66 observations)		China (95 observations)	
	Null model	GNC model	Null model	GNC model	Null model	GNC model
*β*	1.09	0.96	1.13	0.93	1.134	0.81
	[1.02, 1.17]	[0.86, 1.05]	[1.07, 1.20]	[0.83, 1.02]	[0.98 1.31]	[0.67, 0.96]
*α*		0.21		0.23		0.33
		[0.11, 0.30]		[0.14, 0.32]		[0.25, 0.40]
Adj $R^{2}$	0.889	0.908	0.952	0.967	0.675	0.815
AIC	30.3	13.9	−5.97	−27.7	123	70.5
BIC	35.42	21.5	−1.59	−21.2	128	78.1

### Comparing mechanisms: the case of homicide rates

As mentioned earlier, both inter- and intra-city network effects are likely to contribute to superlinear scaling relationships. Recent theories have proposed that the mechanisms underlying urban scaling are the densification of intra-city interactions as cities become larger, leading to exponents around 1.15 for a variety of features (2, 7, 28, 29). Similarly, inter-city interactions could also lead to greater urban outputs. An important mechanism to increase the rate of urban outputs is through bringing together complementarities—individuals with different skills. Different degrees of complementarities required have been successful in explaining the different scaling exponents observed for urban outputs (12, 13). Consequently, the kinds of interaction driving urban outputs may differ across the kinds of outputs, depending on the kind of interactions that are relevant. Since global firms are important participants in bringing together complementarities from other cities to drive economic outputs, we expect GDP to be affected by connection with other cities.

**Fig. 3.**
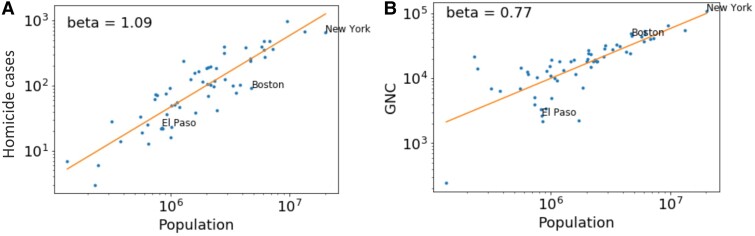
(A) The scaling of homicide cases in US cities. (B) The scaling of GNC for the same cities in the dataset. Selected cities are labeled for illustration.

Thus, to understand the mechanisms at play, we need a way to disentangle local vs. global network effects. Previous work has already shown how the mechanisms of intra-city networks can be tested by looking at features that should or should not depend on team interactions (13). Specifically, crimes that require groups of people have superlinear scaling, and crimes that can be committed as individuals follow linear scaling (13). We can apply this same sort of reasoning to understand inter-city mechanisms by considering features that would benefit from increased interactions and those that are primarily local. Homicide cases are known to scale superlinearly with city population (2, 13), and the kind of interactions that lead to it should be predominantly local, such as in peer groups (30). Thus, homicide cases provide an ideal test case for superlinear scaling that should be largely independent of the between-city firm network effects that we have already argued should enhance GDP. For this purpose, we analyzed the number of homicide cases in the United States in place of GDP, using the same two methods (see Supplementary material for data sources). The scaling of homicide cases and GNC for cities in the dataset are shown in Fig. 3.

We repeat the residual analysis and find the Pearson correlation between the scaling residual for the number of homicide instances, and that of GNC is −0.21, with a *P*-value of 0.11. The correlation is not statistically significant. We also repeat the regression analysis and find the GNC coefficient is not significantly different from 0, and the GNC model is no better description of the data compared to the null model. The regression results are summarized in Table. 3. These results confirm that GNC does *not* play a significant role on the superlinear scaling of homicide instances with city population, and our previous results on GDP are not an artificial consequence of our analysis method.

**Table 3.** Estimated coefficients, 95% CI, and model evaluation metrics for US homicide data.

	Null model	GNC model
*β*	1.09 [0.94, 1.24]	1.23 [1.00, 1.46]
*α*		−0.18 [−0.42, 0.05]
Adj R–sq	0.78	0.79
AIC	107.47	106.8
BIC	111.62	113.1

These results reinforce the possibility that the superlinear scaling of GDP is in part driven by the connection between cities. However, it is important to note that to fully uncover the mechanisms driving GDP future work will need to compare the magnitude of these effects in the same system of units. That is, are the GNC contributions of comparable dollar amounts to the endogenous GDP contributions to a city’s total GDP? This is beyond the scope of the data analyzed here.

## Discussion

Here, we put forward the hypothesis that global inter-city organizational connection plays an important role in the superlinear scaling of urban economic indicators. Our analysis of 255 urban areas in EU, United States, and China provides preliminary support for this hypothesis. Our result suggests that the same increasing return to scale can be phenomenologically explained by the connections between cities as well as, if not better than, the social connections within cities. While our analysis demonstrates that inter-city organizational connections are associated with GDP, our method cannot draw conclusions about causal relationships—whether cities are more prosperous because they are better connected or are they better connected because they are more prosperous, or something else that causes both. To further investigate these relationships, it is important for future studies to consider how organizations shape economic interactions within cities and how organizations connect cities globally—and incorporate them into future urban scaling models. While our study includes the maximum number of cities available from the GNC dataset, data availability limits us to a subset of these three urban systems biased toward larger cities. Future studies could benefit from extending data collection to encompass a broader range of cities to enable a more comprehensive analysis of the global urban system.

**Fig. 4.**
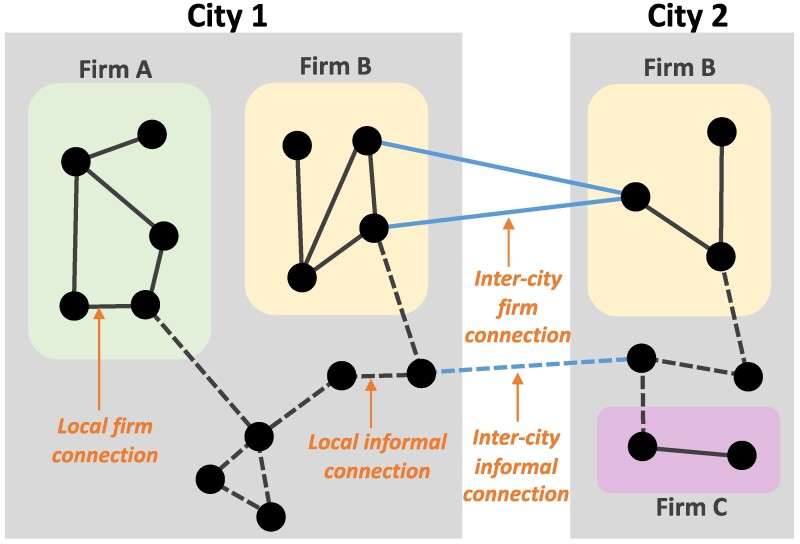
A conceptual figure illustrating nested network of local and inter-city connections.

Various mechanistic proposals for the superlinear scaling of urban indicators are based on the effects of social interactions. Indeed, residents of larger cities have greater sociality, manifested in more phone contacts (11). Whether these social interactions produce economic outputs (such as that can be measured by GDP), and how much, remains an open empirical question. Many previous propositions of urban scaling models have assumed these social interactions to be directly proportional to economic outputs (7, 10). This assumption is at odds with the long-known idea that economic performance is shaped by the kind and the quantity of institutions (25, 26). It should be noted that many types of social connections should be considered if these interaction rates are responsible for superlinear returns to city sizes. Imagine an individual in a city, their interactions are nested in important ways across different collections of people. For example, the individual responsible for producing new ideas or innovations lives in a particular city where they interact with acquaintances, peers in their field, and complete strangers due to the structure of the city. They are also situated within a particular company or university, where their interactions are defined by the structure of that organization. Furthermore, their institution of employment may have strong ties with other branches of the same company in other cities, or may structure the interaction with colleagues at peer institutions in other cities. An example of this nested network is conceptually illustrated in Fig. 4. Thus, the individual who is producing innovations that count toward their home city has a complicated and nested set of interactions that may lead to these innovations, which include their organization, their city of residence, and other cities. How should we weigh each of these connections? How much of their production is due to interaction within their own city compared with interacting with individuals from another city? Why do economic outputs scale superlinearly with a city’s size, and is this because the city’s connectivity with other cities also increases? It has been noted that the local and global processes interact (18), and future research would benefit from weighing the importance of the nested set of connections to answer these questions.

While more populated cities lead to greater economic prosperity, recent research in urban scaling has noted that this greater prosperity is unevenly distributed—the economic benefit of being in larger cities is only manifested among the wealthier individuals (31, 32). Our inter-city organization connection hypothesis may be helpful in explaining these observations—since organizations such as firms and universities are important places allowing for interactions that generate economic outputs, individuals associated with more resourceful, more globally connected organizations gain benefits while those who are not connected are left worse off. Future research can benefit from investigating how connection with global organizations could explain economic inequality in urban areas.

Our work offers several contributions to the literature. Firstly, it challenges the dominant theories that prioritize intra-city connections by highlighting the significance of inter-city ties in economic outputs. Among the few studies that have explored inter-city effects, ours uniquely integrates the influence of organizational connections, an aspect often overlooked despite its acknowledgment in other disciplines. Finally, it spotlights the role of both inter- and intra-city interactions to catalyze a reconsideration of mechanisms leading to the superlinear scaling of urban outputs. By examining GDP and homicide rates, we show the differential impacts of global connectivity on urban outcomes. While economic performance, represented by GDP, correlates with broader city-to-city connections, phenomena like homicides remain predominantly influenced by internal city dynamics. This differentiation suggests that a city’s economic outputs and certain socio-behavioral patterns may not be universally attributed to the same mechanisms. The study, thus, establishes a foundation for more comprehensive urban scaling models that incorporate both intra and inter-city considerations. These future models could consider distinctions among types of social connections that are of different economic value (for example, between individuals and those through organizations), inter-city connections, and the heterogeneity of the different connections within a city. They would consider the interaction between social processes that occur locally within a city, and the interactions happening across cities, especially through economic institutions. Previous empirical studies have focused on either one, while few have studied the interaction between the two (18). Developing a quantitative, predictive theory capturing such interactions would be essential for a more complete theory of urban scaling that explains the new phenomena.

## Supplementary Material

pgae503_Supplementary_Data

## Data Availability

The data analyzed, and the code used to perform our analysis are available online at: https://github.com/vc-yang/global_cities.

## Preprints

This manuscript was posted on a preprint: https://papers.ssrn.com/sol3/papers.cfm?abstract_id=4607355.
