# Supplementary material for: Inter-city firm connections and the scaling of urban economic indicators
Source: PNAS Nexus. 2024 Nov 9;3(11):pgae503. doi: 10.1093/pnasnexus/pgae503 (PMC11579658; doi:10.1093/pnasnexus/pgae503)
Supplement: pgae503_Supplementary_Data [file pgae503_supplementary_data.pdf]

Inter-city firm connections and the scaling of urban economic indicators

Data Sources

The global network connectivity data uses are accessed through the World City Network 2016: Service Value Matrix and Global Network Connectivities, from https://www.lboro.ac.uk/microsites/geography/gawc/datasets/da28.html, accessed in January 2021. The metric for city connectivity used in our analysis is the absolute global network connectivity (GNC). GNC uses the co-presence of 175 global firms, which are in the industry of accountancy, advertising, financial services, law, and management consultancy to construct a network of 707 cities in the world.

The GDP and population data for Chinese cities are obtained through All China Data Center https://www.china-data-online.com/member/city/, accessed in February 2021. Both GDP and population are obtained for prefecture-level cities for year 2016. The data service requires a subscription to access. The population metric used is the estimated permanent resident (Changzhu) population, which measures the amount of people living in a city for sixed months of longer at the time of the survey. The estimates are based on permanent resident population reported for prefecture-level cities in the census every ten years.

The GDP data for US MSAs for year 2016 are obtained from the Bureau of Economic Analysis of the United States, openly available at https://www.bea.gov/news/2017/gross-domestic-product-metropolitan-area-2016. The population data for US MSAs are obtained from the US Census Bureau, available at https://www.census.gov/data/tables/time-series/demo/popest/2010s-total-metro-and-micro-statistical-areas.html. The data sources were accessed in January 2021.

The GDP and population data for cities in the European Union are obtained from the OECD, available at https://stats.oecd.org/Index.aspx?DataSetCode=CITIES. The data measures resident population, and the definition for cities are Functional Urban Areas.

The data for homicide cases in the US are the number of instances of crime under “murder and nonnegligent manslaughter" from the US Federal Bureau of Investigation, Uniform Crime Reports 2016, Table 4, Crime in the United States by Metropolitan Statistical Area. The dataset is accessed from https://ucr.fbi.gov/crime-in-the-u.s/2016/crime-in-the-u.s.-2016/topic-pages/tables/table-4.

Code and data availability

The code and data used to perform our analysis can be found in the following Github Repository: https://github.com/vc-yang/global_cities.
